# Effects of the Water/Cement Ratio on the Properties of 3-3 Type Cement-Based Piezoelectric Composites

**DOI:** 10.3390/ma15082760

**Published:** 2022-04-08

**Authors:** Jian-hong Wang, Hao-xin Sun, Ying-ge Dong, Zhi Cheng, Wei Liu

**Affiliations:** School of Materials Science and Engineering, North University of China, Taiyuan 030051, China; wangjianhong@nuc.edu.cn (J.-h.W.); sunhaoxin@mail.tsinghua.edu.cn (H.-x.S.); bcf1979@163.com (Y.-g.D.); etapple163@163.com (Z.C.)

**Keywords:** 3-3 type cement-based piezoelectric composites, water/cement ratio, microstructure, electromechanical properties, electrical properties

## Abstract

In this work, 3-3 type porous lead zirconate titanate (PZT) ceramics were fabricated by incorporating particle-stabilized foams using the gel-casting method. Then, Portland cement pastes with different water/cement ratios (w/c) were cast into the porous ceramics to produce cement-based piezoelectric (PZT-PC) composites. The effects of w/c on phase structure, microscopic morphology, and electrical properties were studied. The results showed that the amount of hydrated cement products and the density of the PZT–PC composites increased with the increase of w/c from 0.3 to 0.9 and then decreased till w/c achieved a value of 1.1. Correspondingly, the values of both *ε*_r_ and *d*_33_ increased with the density of the PZT–PC composites, resulting in less defects and greater poling efficiency. When w/c was maintained at 0.9, the 3-3 type cement-based piezoelectric composites presented the greatest *K*_t_ value of 40.14% and the lowest *Z* value of 6.98 MRayls, becoming suitable for applications in civil engineering for structural health monitoring.

## 1. Introduction

For many important civil engineering structures and infrastructures, such as high-rise buildings, large-span bridges, and large-scale stadiums, defects and deterioration can appear with time as a consequence of fatigue loading, environmental corrosion, and material aging during their long-term service. Therefore, it is very important to employ a civil structural health monitoring system to diagnose the presence of defects and the extent of damage in real time, which can help the timely repair and reinforcement of civil engineering structures, thus ensuring their integrity and safety [1,2,3,4,5].

It is known that a structural health monitoring system is mainly composed of sensor and actuator units, which employ smart materials to realize signal receiving and driving. Typical smart materials are piezoelectric ceramics, piezoelectric polymer, shape-memory alloys, and optical fibers [6,7,8]. Based on the piezoelectric effect and electromechanical coupling effect, piezoelectric ceramic transducers, which usually utilize lead zirconate titanate (PZT) ceramic as the critical raw material, are able to excite and receive signals and have become the most commonly used components of structural health monitoring (SHM) systems [9,10,11,12]. However, as the acoustic impedance of PZT ceramics approximates 30 MRayls, a value much higher than that of concrete (~9 MRayls), the compatibility problem between piezoelectric ceramic transducer and civil engineering structure tends to cause obvious excitation signal loss and a deterioration of the measurement accuracy [13,14,15]. Therefore, cement-based piezoelectric composites were invented using cement as the matrix and piezoelectric ceramics as the functional component to close the gap of acoustic impedance (*Z*) between piezoelectric transducers and cements [16,17,18,19]. For example, Li et al. fabricated 0-3 type cement-based piezoelectric composites by mixing white Portland cement with PZT ceramic powders, which successfully decreased the acoustic impedance (*Z*) to about 8.95 MRayls and resolved the compatibility problems [20]. However, it should be noted that, as PZT ceramic powders are dispersed in the three-dimensionally connected cement matrix, it is difficult to obtain excellent piezoelectric properties for 0-3 type cement-based piezoelectric composites, due to the poor poling efficiency. One optional way to improve the degree of poling is to introduce a small volume of conductive component, such as carbon black or nanotubes, to form a continuous electric network between the piezoelectric particles, but the dielectric loss and energy dissipation of the composites will increase remarkably with the consequent decrease in resistivity [21,22,23,24].

Our group, recently, fabricated 3-3 type cement-based piezoelectric composites by casting cement paste into 3-3 type porous PZT ceramics. The porous ceramics were prepared from particle-stabilized ceramic foams via the gel-casting technique. The connection of the piezoelectric components made it possible for the 3-3 type composites to improve the degree of poling and the piezoelectric properties [25]. Nonetheless, few studies have been carried out to comprehend the effects of the water/cement ratio in the cement paste on the properties of 3-3 type PZT-PC composites. In the present work, we investigated the effect of the water/cement ratio in the cement paste on the structural, electrical, and electromechanical properties of cement-based piezoelectric composites, such as phase structure, microscopic morphology, acoustic impedance, electromechanical coupling coefficients, etc.

## 2. Experimental

As mentioned above, the preparation procedure of 3-3 type cement-based piezoelectric composites can be divided into two steps. First, a ceramic slurry with a solid loading of 15 vol% and a valeric acid concentration of 70 mmol/L was prepared by ball-milling the two raw materials in a premix solution (water/acrylamide (AM, C_2_H_3_CONH_2_)/ *N*, *N*′-methylenebisacrylamide (MBAM, (C_2_H_3_CONH)_2_CH_2_) = 85: 14.5: 0.5), then the slurry was mechanically stirred to generate foams, whilst the catalyst (*N, N, N′, N′*-tetramethylenediamine) and the initiator (ammonium persulfate, (NH_4_)_2_S_2_O_8_, 35 wt%) were added to induce the polymerization. Afterwards, the green body was air-dried and sintered at 1150 °C for 2 h to obtain 3-3 type porous PZT ceramics. In the second step, the Portland cement paste with different water/cement ratios (0.3, 0.5, 0.7, 0.9, 1.1) was cast in the porous PZT ceramics and cured in the presence of moisture at 20 °C, with 100% RH for 28 days to acquire the 3-3 type cement-based piezoelectric composites.

The rheological properties of the cement paste were measured with a viscometer Brookfield DV-II +Pro. The morphology of the composites was characterized by scanning electron microscopy (Hitachi, SU-5000, Tokyo, Japan). The XRD patterns were recorded using a Rigaku D/MAX-2400 X-ray diffractometer. The density of the composites was studied using the Archimedean method. For the electrical measurements, the composites were coated with low-temperature silver paint, and the polling process was carried out in a silicon oil bath under a dc field of 3 kV/mm at 120 °C for 30 min. The longitudinal piezoelectric coefficient (*d*_33_) was measured by *d*_33_ m (ZJ-3A, Institute of Acoustics, Chinese Academy of Science, Beijing, China). The dielectric properties of the composites were collected using an HP4194 impedance analyzer. The impedance characteristics of the samples were investigated in the frequency range of 0–1000 kHz at room temperature. The ferroelectric hysteresis (*P*–*E*) loops measurement at 100 Hz was carried out by a Radiant Technologies RT6000HVA test system (Radiant Technologies, Inc., Albuquerque, NM, USA).

The thickness electromechanical coupling coefficient (*K*_t_) was approximated from the following formula:

(1) $$K_{t}^{2}=\frac{\pi }{2}\cdot \frac{f_{s}}{f_{p}}\cdot tan\left(\frac{\pi }{2}\cdot \frac{f_{p}-f_{s}}{f_{p}}\right)$$

The planar electromechanical coupling coefficients (*K*_p_) can be evaluated using the curve of *K*_p_ versus Δ*f*/*f*_s_.

The acoustic impedance (*Z*) can be obtained from the following equation:

(2) $$Z=2\cdot \rho_{c}\cdot d\cdot f_{p}$$

where *f*_s_ and *f*_p_ are the series resonant frequency and the parallel resonant frequency, respectively, which can be replaced by the frequencies at the minimum and maximum impedances (*f*_m_ and *f*_n_) in the fundamental resonant region of the impedance spectrum; *d* and *ρ*_c_ are the thickness and density of the PZT–PC composites, respectively.

## 3. Results and Discussion

### 3.1. Rheological Behavior of the Cement Paste

**Figure 1:**
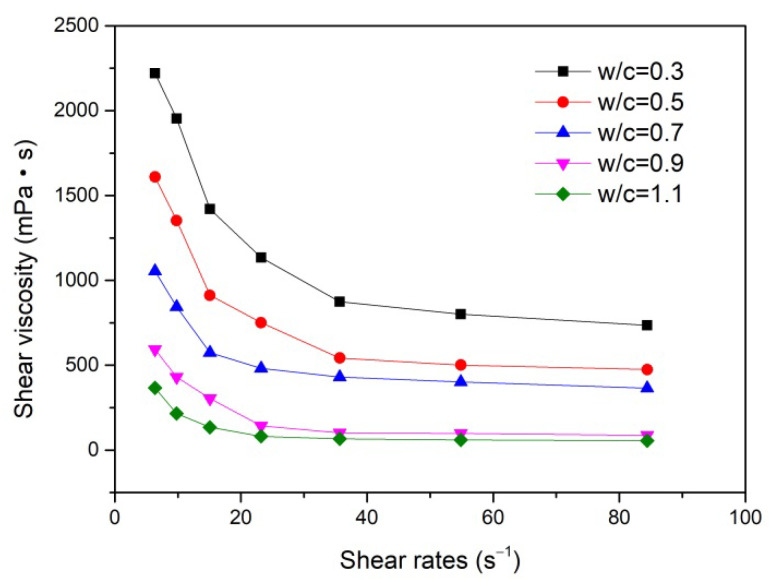
Rheological behavior of the cement paste with different water/cement ratios.

Figure 1 depicts the apparent viscosity of the cement paste versus the water/cement ratio. As expected, the apparent viscosity demonstrated a shear thinning behavior within the applied shear rate range and a distinct decreasing tendency with the rise of w/c from 0.3 to 1.1. It was noted that the rheology behavior of the cement paste was basically determined by physical-chemical inter-particle and particle–medium interactions, which depended not only on the nature and composition of the cement particles, but also on the water/cement ratio of the cement paste. Therefore, with the increase in water/cement ratio, the range of inter-particle repulsion increased sufficiently with lower van der Waals attraction and thus prevented the cement particles to agglomerate, which rendered the cement hydration products easy to fluctuate and promoted the infiltration of the cement components into the porous PZT ceramics.

### 3.2. Phase Analysis and Microstructure

**Figure 2:**
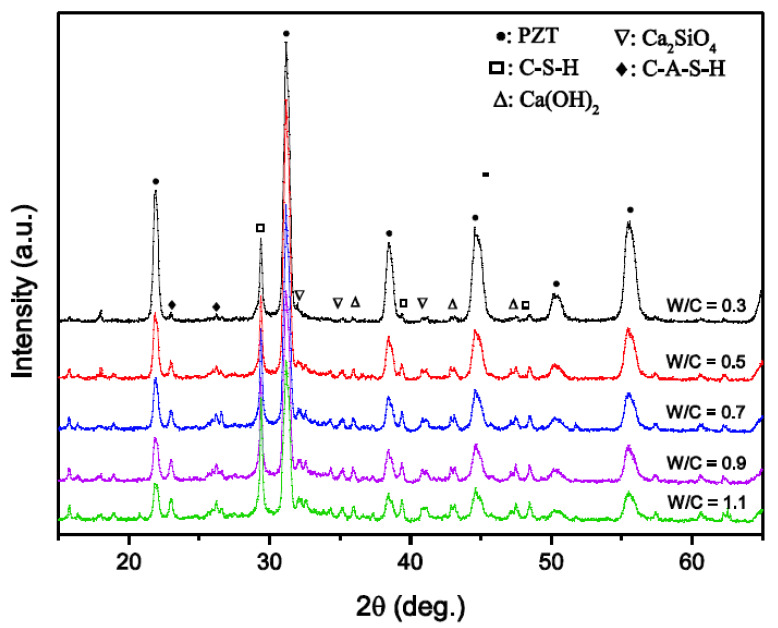
XRD patterns of PZT–PC composites with different water/cement ratios.

Figure 2 shows the influence of the water/cement ratio on the X-ray diffraction patterns of the PZT–PC composites. It can be seen that, due to the relatively greater amount of PZT base material in the composites, the dominant diffraction peaks around 21.8°, 31.2°, 38.5°, 44.6°, and 55.4° corresponded to the typical perovskite phase of PZT, independently of the water/cement ratio of the cement paste. Moreover, the crystalline peaks of cement hydration products, such as calcium silicate hydrates (C–S–H), calcium hydroxide (Ca(OH)_2_), dicalcium silicates (2CaO·SiO_2_, shown as Ca_2_SiO_4_), and ettringite (C–A–S–H), could be detected, and the diffraction intensity increased notably as the w/c of the cement paste increased from 0.3 to 0.9, which confirmed that the quantity of cement hydration products combined with porous PZT ceramics increased with a decreasing viscosity of the cement paste. However, it should be noted that, while the w/c increased to 1.1, the diffraction peak intensity of cement hydration was not further enhanced, implying a decreased combined quantity in spite of the decreasing viscosity of the cement paste.

**Figure 3:**
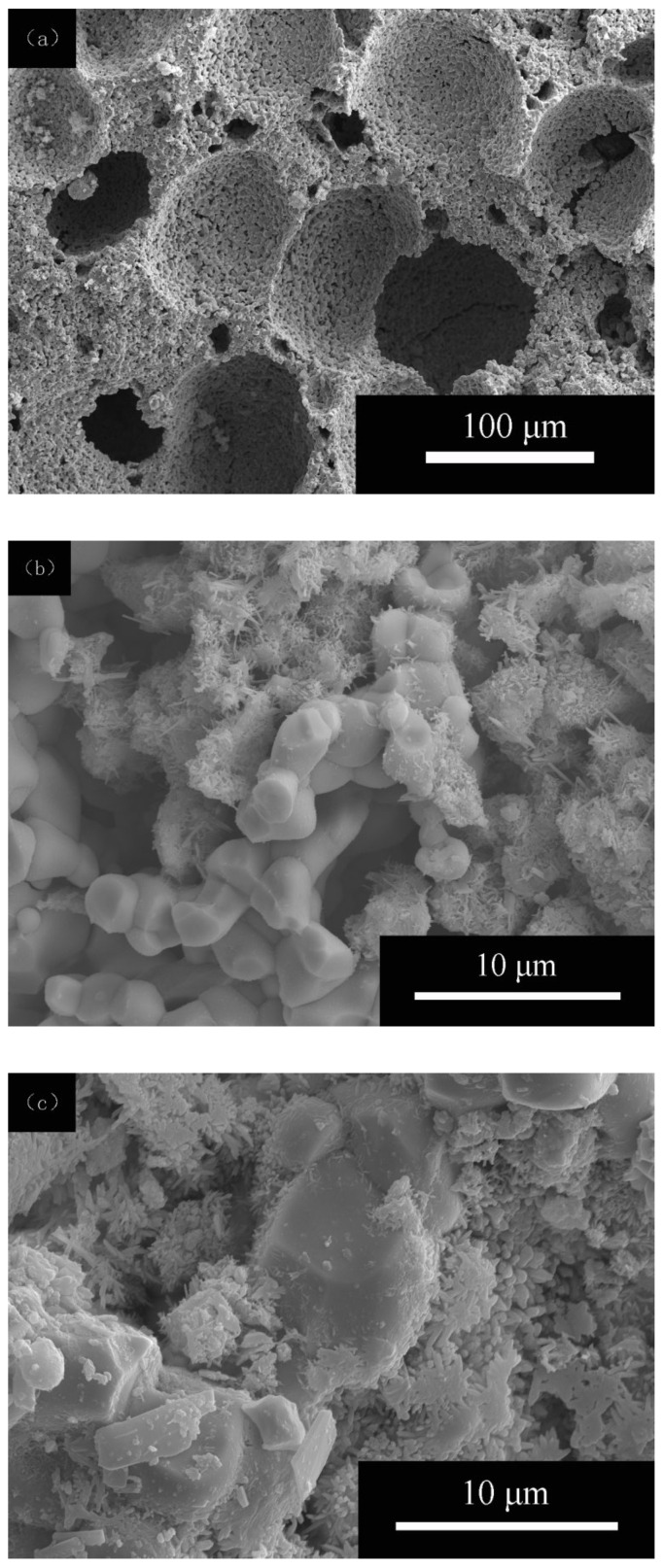

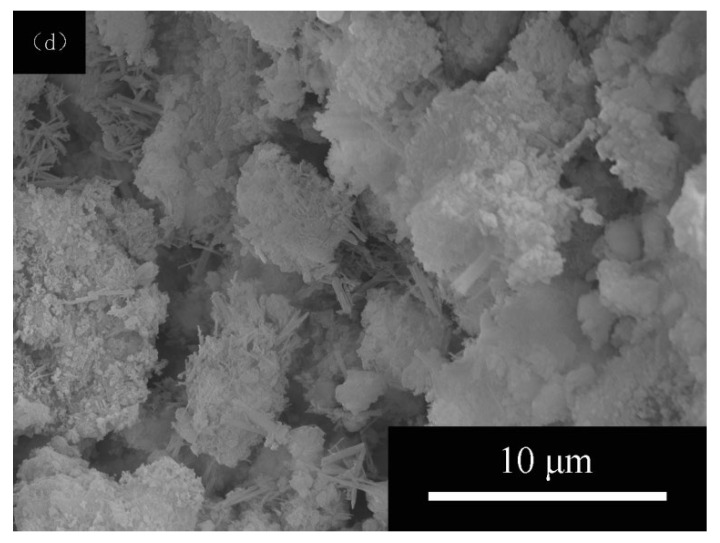
Scanning electron micrographs of (**a**) porous PZT ceramics and PZT–PC composites with water/cement ratios of (**b**) 0.5; (**c**) 0.7; (**d**) 0.9.

The detailed pore geometry and interconnection of porous PZT ceramics and PZT–PC composites are shown in Figure 3. As can be seen in Figure 3a, porous ceramics with open cells maintained a uniform pore size distribution due to the low solid loading of the ceramic slurry, which was convenient for the penetration of the cement paste into the porous ceramics. As depicted in Figure 3b,c, when the cement paste was introduced in the porous PZT ceramics, the interface bonding between PZT ceramic particles and cement hydration products was quite evident, and the filling degree of the pore space of the PZT ceramics by the cement hydration products increased with the water/cement ratio. As shown in Figure 3d, when w/c increased to 0.9, the PZT ceramic particles were almost surrounded by cement hydration products such as small fibrous crystals, characteristic of calcium silicate hydrates (C–S–H), and long-needle-shaped ettringite crystals (C–A–S–H), which allowed closer connections between the solid particles and the symmetrical structure, increasing its strength and stiffness.

### 3.3. Dielectric and Piezoelectric Properties

**Figure 4:**
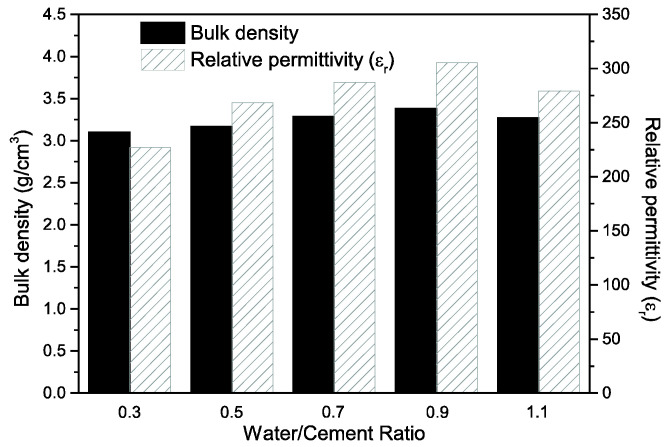
Variation of density and relative permittivity (*ε*_r_) of PZT–PC composites with different water/cement ratios.

Figure 4 shows the variation of density and relative permittivity (*ε*_r_) of the PZT–PC composites in relation to the water/cement ratio of the cement paste. As discussed above, the viscosity of the cement paste decreased with the increase of the water/cement ratio, which made it easier for the cement paste to penetrate into the porous PZT ceramics. Therefore, when the w/c increased from 0.3 to 0.9, the density of the composites correspondingly increased from 3.10 g/cm^3^ to 3.38 g/cm^3^. However, it must be noted that the density of the composites decreased to 3.27 g/cm^3^ when the w/c reached 1.1, which implied that the excessively low solid content of the cement paste led to a reduction of cement hydration products, despite the presence of more cement paste in the holes of the porous PZT ceramics with low viscosity. With the change in density, the relative permittivity of the PZT–PC composites varied between 227.22 and 305.24. It could be deduced that the PZT–PC composites with high density possessed less defects and higher polarization efficiency owing to the substitution of the cement components by air in the pores, and this was beneficial to the movement of domain walls and the enhancement of *ε*_r_ values [26]. Moreover, the *ε*_r_ of the 3-3 type composites was much greater than that of the 0-3 type composites, which indicated that the excellent interaction of the PZT phase with the 3-3 type composites contributed to the flow of electric current [1].

**Figure 5:**
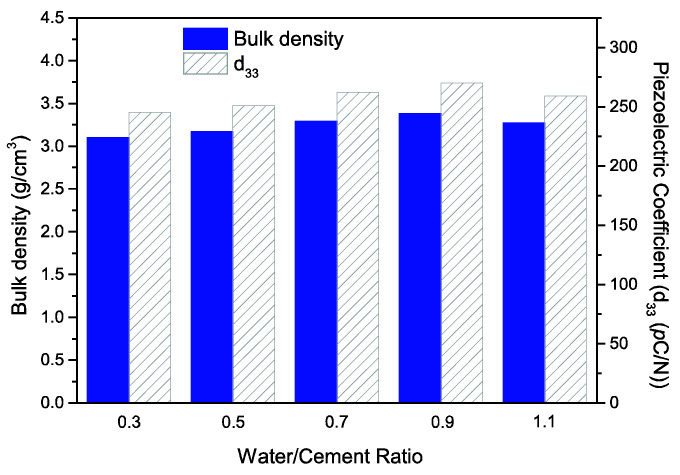
Variation of density and longitudinal piezoelectric coefficient (*d*_33_) of the PZT–PC composites at different water/cement ratios.

**Figure 6:**
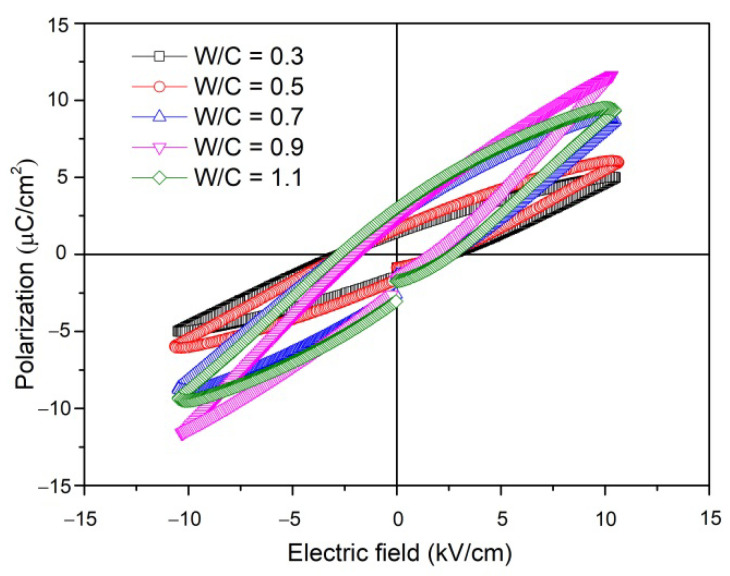
*P*–*E* hysteresis loops of the PZT–PC composites vs. water/cement ratio.

The longitudinal piezoelectric strain coefficient (*d*_33_) of the PZT–PC composites as a function of the water/cement ratio is depicted in Figure 5. Similar to the varying tendency of the relative permittivity (*ε*_r_), the value of *d*_33_ increased from 245 *p*C/N to 270 *p*C/N for w/c ranging from 0.3 to 0.9 and decreased to 259 *p*C/N until w/c reached 1.1; this value was much higher than that found for 0-3 type PZT–PC composites produced by others previously [20,27]. As is well known, the poling electric field is the driving force that determined the switch and reorientation of the ferroelectric domain in the PZT component and imparts piezoelectric properties to the PZT–PC composites. First of all, the interconnection of the PZT component in the 3-3 type composites helped the formation of a continuous electric flux path on the PZT particles and facilitated the domain motion reorientation, thus leading to the enhancement of the piezoelectric properties. Secondly, by combining porous PZT ceramics with cement hydration products, the increase in the density of the composites eliminated the inner holes accordingly, which would weaken the depolarization field during the poling process and improve the poling efficiency of the piezoelectric active component [28]. The polarization–electric field (*P*–*E*) hysteresis loops were analyzed to further investigate the effect of the water/cement ratio on the piezoelectric properties of PZT–PC composites. As can be seen in Figure 6, incompletely saturated *P*–*E* hysteresis loops with elliptical shape were acquired at room temperature for all specimens, which could indicate that the existence of nonpiezoelectric hydrated cement products inhibited the polarization of the PZT component. Besides, the “lossy” feature of the *P*–*E* hysteresis loops should not be neglected. It appeared in the form of an evident gap in the negative remanent polarization and is believed to be caused by weak conducting ions such as Al^3+^, Ca^2+^, and OH^−^ in the cement matrix, which migrated in alignment with the piezoelectric domain of the PZT component and brought about a certain degree of conducting losses during the polarization process [29]. Moreover, both the coercive field (*E*_c_) and the remnant polarization (*P*_r_) of the composites changed with the introduction of the cement component and reached the maximum values of 1.92 kV/cm and 2.31 μC/cm^2^ for a water/cement ratio of 0.9, which was nearly the same as that of the *d*_33_ values.

### 3.4. Electromechanical and Acoustic Properties

The electromechanical coupling coefficients (*K*_t_, *K*_p_) and acoustic impedance (*Z*_c_) of the PZT–PC composites are shown in Table 1. It is seen that the *K*_t_ value increased from 28.77% to 40.14% with the increase of w/c from 0.3 to 0.9; thereafter, the *K*_t_ value decreased to 36.56% for a w/c of 1.1. The *K*_p_ value changed identically between 28.54% and 36.96%, while the *K*_t_ of the 0-3 type composites prepared by Li et al. ranged from 11.6% to 20.7% [1]. In any case, the higher value of *K*_t_ with respect to that of *K*_p_ indicated enhanced thickness mode oscillations and is helpful for structural health monitoring. Meanwhile, it was found that both the thickness and the planar mode vibrations of the PZT–PC composites were strengthened by compounding with cement and could be tailored by adjusting the combined quantity of the cement component.

Regarding the acoustic impedance (*Z*), it did not change with the density of the PZT–PC composites but demonstrated an entirely opposite trend in relation to the water/cement ratio or the corresponding densities of the PZT–PC composites. The value of acoustic impedance (*Z*) decreased with the increase of the water/cement ratio and reached the minimum value of 6.98 MRayls at a w/c of 0.9, which could be ascribed to the obvious decrease of the parallel resonant frequency (*f*_p_) with the enhancement of the poling efficiency following the introduction of the cement component. In any case, the acoustic impedance (*Z*) of the composites was almost equal to that of concrete structures (~6.9–11.23 MRayls) and beneficial to improve the acoustic matching in civil engineering application.

## 4. Conclusions

In the present research, 3-3 type cement-based piezoelectric composites were fabricated by casting a cement paste into porous PZT ceramics. By changing the water/cement ratio, both the viscosity of the cement paste and the amount of the cement component combined with the piezoelectric ceramics were adjusted. When increasing the w/c from 0.3 to 0.9, the decreasing viscosity of the cement paste contributed to the infiltration of the cement component into the inner parts of the porous PZT ceramics and increased the density of the PZT–PC composites from 3.10 g/cm^3^ to 3.38 g/cm^3^. When the w/c achieved 1.1, the density of the composites decreased to 3.27 g/cm^3^, reducing the amount of hydration cement products. As for the dielectric and piezoelectric properties of the PZT–PC composites, the relative permittivity *ε*_r_ and the piezoelectric strain coefficient *d*_33_ increased with the density of the composites due to less defects and higher polarization efficiency. When the water/cement ratio was maintained at 0.9, the 3-3 type cement-based piezoelectric composites acquired the greatest *K*_t_ value of 40.14%, higher than the maximum *K*_p_ value of 36.96%, which implied an enhanced thickness mode oscillation for the composites. The Z values of the PZT–PC composites varied between 6.98 MRayls and 7.19 MRayls, values which were as low as those of a cement matrix and may be useful in civil structural health monitoring applications.

## Figures and Tables

**Table 1:** Electromechanical and acoustic properties of the PZT–PC composites with different water/cement ratios.

Water-to-Cement Ratio	0.3	0.5	0.7	0.9	1.1
*K*_p_/%	26.32	27.23	30.02	34.57	36.93
*K*_t_/%	26.75	30.02	31.01	35.25	34.46
*Z*/MRayls	7.19	7.13	7.13	6.98	7.09

## Data Availability

All the data is available within the manuscript.
